# PPF Dependent Fixed Point Results for Triangular *α*_*c*_-Admissible Mappings

**DOI:** 10.1155/2014/673647

**Published:** 2014-02-02

**Authors:** Ljubomir Ćirić, Saud M. Alsulami, Peyman Salimi, Pasquale Vetro

**Affiliations:** ^1^Faculty of Mathematics Engineering, Kraljice Marije 16, 11 000 Belgrade, Serbia; ^2^Department of Mathematics, King Abdulaziz University, P.O. Box 80203, Jeddah 21589, Saudi Arabia; ^3^Young Researchers and Elite Club, Rasht Branch, Islamic Azad University, Rasht, Iran; ^4^Università degli Studi di Palermo, Dipartimento di Matematica e Informatica, Via Archirafi 34, 90123 Palermo, Italy

## Abstract

We introduce the concept of triangular *α*_*c*_-admissible mappings (pair of mappings) with respect to *η*_*c*_ nonself-mappings and establish the existence of PPF dependent fixed (coincidence) point theorems for contraction mappings involving triangular *α*_*c*_-admissible mappings (pair of mappings) with respect to *η*_*c*_ nonself-mappings in Razumikhin class. Several interesting consequences of our theorems are also given.

## 1. Introduction and Preliminaries

The applications of fixed point theory are very important and useful in diverse disciplines of mathematics. In fact, fixed point theory can be applied for solving equilibrium problems, variational inequalities, and optimization problems. In particular, a very powerful tool is the Banach fixed point theorem, which was generalized and extended in various directions: modifying Banach's contractive condition, changing the space, or extending single-valued mapping to multivalued mapping (see [1–8] and references therein). In 1997, Bernfeld et al. [9] introduced the concept of fixed point for mappings that have different domains and ranges, which is called PPF dependent fixed point or the fixed point with PPF dependence. Furthermore, they gave the notion of Banach type contraction for nonself-mapping and also proved the existence of PPF dependent fixed point theorems in the Razumikhin class for Banach type contraction mappings (also see [10]). The PPF dependent fixed point theorems are useful for proving the solutions of nonlinear functional differential and integral equations which may depend upon the past history, present data, and future consideration. On the other hand, Samet et al. [11] first introduced the concept of *α*-admissible self-mappings and proved the existence of fixed point results using contractive conditions involving *α*-admissible mappings in complete metric spaces. They also gave some examples and applications of the obtained results to ordinary differential equations. In this paper, we will introduce the concept of triangular *α*_*c*_-admissible mappings (pair of mappings) with respect to *η*_*c*_ nonself-mappings and establish the existence of PPF dependent fixed point theorems for contraction mappings involving triangular *α*_*c*_-admissible mappings (pair of mappings) with respect to *η*_*c*_ nonself-mappings in Razumikhin class.

Throughout this paper, we assume that (*E*, ||·||_*E*_) is a Banach space, *I* denotes a closed interval [*a*, *b*] in ℝ, and *E*_0_ = (*I*, *E*) denotes the sets of all continuous *E*-valued functions on *I* equipped with the supremum norm ||·||_*E*_0__ defined by

(1) $$\begin{array}{c} {||\phi ||}_{E_{0}}=\underset{t\in I}{\sup}{||\phi \left(t\right)||}_{E}. \end{array}$$

For a fixed element *c* ∈ *I*, the Razumikhin or minimal class of functions in *E*_0_ is defined by

(2) $$\begin{array}{c} {ℛ}_{c}=\left\{\phi \in E_{0}:{||\phi ||}_{E_{0}}={||\phi \left(c\right)||}_{E}\right\}. \end{array}$$

Clearly, every constant function from *I* to *E* belongs to *ℛ*_*c*_.

**Definition 1**

Let *ℛ*_*c*_ be the Razumikhin class; then

- the class *ℛ*_*c*_ is algebraically closed with respect to difference, if *ϕ* − *ξ* ∈ *ℛ*_*c*_ when *ϕ*, *ξ* ∈ *ℛ*_*c*_;
- the class *ℛ*_*c*_ is topologically closed if it is closed with respect to the topology on *E*_0_ generated by the norm ||·||_*E*_0__.

**Definition 2 (see [9])**

A mapping *ϕ* ∈ *E*_0_ is said to be a PPF dependent fixed point or a fixed point with PPF dependence of mapping *T* : *E*_0_ → *E* if *Tϕ* = *ϕ*(*c*) for some *c* ∈ *I*.

**Definition 3 (see [10])**

Let *S* : *E*_0_ → *E*_0_ and *T* : *E*_0_ → *E*. A point *ϕ* ∈ *E*_0_ is said to be a PPF dependent coincidence point or a coincidence point with PPF dependence of *S* and *T* if *Tϕ* = (*Sϕ*)(*c*) for some *c* ∈ *I*.

**Definition 4 (see [9])**

The mapping *T* : *E*_0_ → *E* is called a Banach type contraction if there exists *k* ∈ [0,1) such that

(3) $$\begin{array}{c} {||T\phi -T\xi ||}_{E}\leq k{||\phi -\xi ||}_{E_{0}}, \end{array}$$

for all *ϕ*, *ξ* ∈ *E*_0_.

In 2012, Samet et al. [11] introduced the concepts of *α*-*ψ*-contractive and *α*-admissible mappings and established various fixed point theorems for such mappings in complete metric spaces. Afterwards, Karapinar and Samet [12] generalized these notions to obtain fixed point results. More recently, Salimi et al. [13] modified the notions of *α*-*ψ*-contractive and *α*-admissible mappings and established fixed point theorems which are proper generalizations of the recent results in [11, 12].

Samet et al. [11] defined the notion of *α*-admissible mappings as follows.

**Definition**

Let *T* be a self-mapping on *X* and let *α* : *X* × *X* → [0, +*∞*) be a function. We say that *T* is an *α*-admissible mapping if

(4) $$\begin{array}{c} x,y\in X,\;\alpha \left(x,y\right)\geq 1⟹\alpha \left(Tx,Ty\right)\geq 1. \end{array}$$

In [11] the authors consider the family Ψ of nondecreasing functions *ψ* : [0, +*∞*)→[0, +*∞*) such that ∑_*n*=1_^+*∞*^ *ψ* ^*n*^(*t*)<+*∞* for each *t* > 0, where *ψ* ^*n*^ is the *n*th iterate of *ψ* and give the following theorem.

**Theorem 6**

Let (*X*, *d*) be a complete metric space and let *T* be an *α*-admissible mapping. Assume that

(5) $$\begin{array}{c} \alpha \left(x,y\right)d\left(Tx,Ty\right)\leq \psi \left(d\left(x,y\right)\right) \end{array}$$

for all *x*, *y* ∈ *X*, where *ψ* ∈ Ψ. Also, suppose that the following assertions hold:

- there exists *x*_0_ ∈ *X* such that *α*(*x*_0_, *Tx*_0_) ≥ 1,
- either *T* is continuous or for any sequence {*x*_*n*_} in *X* with *α*(*x*_*n*_, *x*_*n*+1_) ≥ 1 for all *n* ∈ *ℕ* ∪ {0} and *x*_*n*_ → *x* as *n* → +*∞*, one has *α*(*x*_*n*_, *x*) ≥ 1 for all *n* ∈ *ℕ* ∪ {0}.

Then *T* has a fixed point.

Salimi et al. [13] modified and generalized the notions of *α*-*ψ*-contractive mappings and *α*-admissible mappings by the following ways.

**Definition 7 (see [13])**

Let *T* be a self-mapping on *X* and *α*, *η* : *X* × *X* → [0, +*∞*) two functions. We say that *T* is an *α*-admissible mapping with respect to *η* if

(6) $$\begin{array}{c} x,y\in X,\;\alpha \left(x,y\right)\geq \eta \left(x,y\right)⟹\alpha \left(Tx,Ty\right)\geq \eta \left(Tx,Ty\right). \end{array}$$

Note that if we take *η*(*x*, *y*) = 1, then this definition reduces to Definition 5. Also, if we take *α*(*x*, *y*) = 1, then we say that *T* is an *η*-subadmissible mapping.

The following result was proved by Salimi et al. [13].

**Theorem 8 (see [13])**

Let (*X*, *d*) be a complete metric space and let *T* be an *α*-admissible mapping. Assume that

(7) $$\begin{array}{c} x,y\in X,\;\alpha \left(x,y\right)\geq 1⟹d\left(Tx,Ty\right)\leq \psi \left(M\left(x,y\right)\right), \end{array}$$

where *ψ* ∈ Ψ and

(8) M(x,y)=max⁡{d(x,y),d(x,Tx)+d(y,Ty)2,   d(x,Ty)+d(y,Tx)2}.

Also, suppose that the following assertions hold:

- there exists *x*_0_ ∈ *X* such that *α*(*x*_0_, *Tx*_0_) ≥ 1,
- either *T* is continuous or for any sequence {*x*_*n*_} in *X* with *α*(*x*_*n*_, *x*_*n*+1_) ≥ 1 for all *n* ∈ *ℕ* ∪ {0} and *x*_*n*_ → *x* as *n* → +*∞*, one has *α*(*x*_*n*_, *x*) ≥ 1 for all *n* ∈ *ℕ* ∪ {0}.

Then *T* has a fixed point.

Recently Karapinar et al. [14] introduced the notion of triangular *α*-admissible mapping as follows.

**Definition (see [14])**

Let *T* : *X* → *X* and *α* : *X* × *X* → (−*∞*, +*∞*). We say that *T* is a triangular *α*-admissible mapping if

$$\begin{array}{cc} \left(\text{T}1\right) & \alpha \left(x,y\right)\geq 1\;\text{implies}\;\;\alpha \left(Tx,Ty\right)\geq 1,\;\;x,y\in X, \end{array}$$

$$\begin{array}{cc} \left(\text{T}2\right) & \begin{array}{c} \alpha \left(x,z\right)\geq 1\\ \alpha \left(z,y\right)\geq 1 \end{array}\;\text{imply }\alpha \left(x,y\right)\geq 1. \end{array}$$

For more details and applications of this line of research, we refer the reader to some related papers [15–21].

Now, motivated by Salimi et al. [13] and Karapinar et al. [14] (see also [15–21]), we introduce the following notion.

**Definition 10**

Let *c* ∈ *I* and *T* : *E*_0_ → *E*, *α*, *η* : *E* × *E* → [0, +*∞*). We say that *T* is a triangular *α*_*c*_-admissible mapping with respect to *η*_*c*_ if, for *ϕ*, *ξ*, *π* ∈ *E*_0_,

(TC1)α(ϕ(c),ξ(c))≥η(ϕ(c),ξ(c))  ⟹α(Tϕ,Tξ)≥η(Tϕ,Tξ),

(TC2)α(ϕ(c),π(c))≥η(ϕ(c),π(c)),α(π(c),ξ(c))≥η(π(c),ξ(c))imply  α(ϕ(c),ξ(c))≥η(ϕ(c),ξ(c)).

Note that if we take *η*(*x*, *y*) = 1 for all *x*, *y* ∈ *E*, then we say that *T* is a triangular *α*_*c*_-admissible mapping. Also, if we take *α*(*x*, *y*) = 1 for all *x*, *y* ∈ *E*, then we say that *T* is a triangular *η*_*c*_-subadmissible mapping.

**Example 11**

Let *E* = ℝ be a real Banach space with usual norm and let *I* = [0,1]. Define *T* : *E*_0_ → *E* by *Tϕ* = 2*ϕ*(1) for all *ϕ* ∈ *E*_0_ and *α*, *η* : *E* × *E* → [0, +*∞*) by

(9) $$\begin{array}{c} \alpha \left(x,y\right)=\{\begin{array}{cc} x^{2}+y^{2}+\left|x\right|\left|y\right|+1, & \text{if}\;x\geq y,\\ 0, & \text{otherwise}; \end{array} \end{array}$$

*η*(*x*, *y*) = *x* ^2^ + *y* ^2^ + |*x*||*y*| + 1/2. Then *T* is a triangular *α*_*c*_-admissible mapping with respect to *η*_*c*_. Indeed, if *α*(*ϕ*(1), *ξ*(1)) ≥ *η*(*ϕ*(1), *ξ*(1)), then *ϕ*(1) ≥ *ξ*(1) and so 2*ϕ*(1) ≥ 2*ξ*(1). That is, *Tϕ* ≥ *Tξ* which implies *α*(*Tϕ*, *Tξ*) ≥ *η*(*Tϕ*, *Tξ*). Also, if

(10) $$\begin{array}{c} \alpha \left(\phi \left(c\right),\pi \left(c\right)\right)\geq \eta \left(\phi \left(c\right),\pi \left(c\right)\right),\\ \alpha \left(\pi \left(c\right),\xi \left(c\right)\right)\geq \eta \left(\pi \left(c\right),\xi \left(c\right)\right), \end{array}$$

then *ϕ*(*c*) ≥ *π*(*c*) and *π*(*c*) ≥ *ξ*(*c*) and so *ϕ*(*c*) ≥ *ξ*(*c*). That is, *α*(*ϕ*(*c*), *ξ*(*c*)) ≥ *η*(*ϕ*(*c*), *ξ*(*c*)).

The following lemma is necessary later on.

**Lemma 12**

Let *T* be a triangular *α*_*c*_-admissible mapping with respect to *η*_*c*_. Define the sequence {*ϕ*_*n*_} by the following way:

(11) $$\begin{array}{c} T\phi_{n-1}=\phi_{n}\left(c\right); \end{array}$$

for all *n* ∈ *ℕ*, where *ϕ*_0_ ∈ *ℛ*_*c*_ is such that *α*(*ϕ*_0_(*c*), *Tϕ*_0_) ≥ *η*(*ϕ*_0_(*c*), *Tϕ*_0_). Then

(12) α(ϕm(c),ϕn(c))≥η(ϕm(c),ϕn(c)),∀m,n∈ℕ  with  m<n.

**Proof**

Since *T* is a triangular *α*_*c*_-admissible mapping with respect to *η*_*c*_,

(13) α(ϕ0(c),ϕ1(c))=α(ϕ0(c),Tϕ0)≥η(ϕ0(c),Tϕ0)=η(ϕ0(c),ϕ1(c))

and so

(14) $$\begin{array}{c} \alpha \left(\phi_{1}\left(c\right),T\phi_{1}\right)\geq \eta \left(\phi_{1}\left(c\right),T\phi_{1}\right). \end{array}$$

By continuing this process we get,

(15) $$\begin{array}{c} \alpha \left(\phi_{n}\left(c\right),\phi_{n+1}\left(c\right)\right)\geq \eta \left(\phi_{n}\left(c\right),\phi_{n+1}\left(c\right)\right),\;\forall n\in \mathbb{N}. \end{array}$$

Since

(16) $$\begin{array}{c} \alpha \left(\phi_{m}\left(c\right),\phi_{m+1}\left(c\right)\right)\geq \eta \left(\phi_{m}\left(c\right),\phi_{m+1}\left(c\right)\right),\\ \alpha \left(\phi_{m+1}\left(c\right),\phi_{m+2}\left(c\right)\right)\geq \eta \left(\phi_{m+1}\left(c\right),\phi_{m+2}\left(c\right)\right), \end{array}$$

then by ((TC2)) we get *α*(*ϕ*_*m*_(*c*), *ϕ*_*m*+2_(*c*)) ≥ *η*(*ϕ*_*m*_(*c*), *ϕ*_*m*+2_(*c*)). By continuing this process, we get

(17) α(ϕm(c),ϕn(c))≥η(ϕm(c),ϕn(c)),∀m,n∈ℕ  with  m<n.

## 2. Main Results

One of our main theorems is a result of Geraghty type [22] obtained by a modification of the approach in [13]. Let *ℱ* denote the class of all functions *β* : [0, +*∞*)→[0,1) satisfying the following condition:

(18) $$\begin{array}{c} \beta \left(t_{n}\right)⟶1\;\text{implies}\;\;t_{n}⟶0,\;\;\text{as}\;\;n⟶+\infty . \end{array}$$

**Theorem 13**

Let *T* : *E*_0_ → *E*, *α*, *η* : *E* × *E* → [0, +*∞*) be three mappings satisfying the following assertions:

(i) there exists *c* ∈ *I* such that *ℛ*_*c*_ is topologically closed and algebraically closed with respect to difference;
(ii) *T* is a triangular *α*_*c*_-admissible mapping with respect to *η*_*c*_;
(iii) there exists *β* ∈ *ℱ* such that
  (19) α(ϕ(c),ξ(c))≥η(ϕ(c),ξ(c))  ⟹||Tϕ−Tξ||E≤β(||ϕ−ξ||E0)||ϕ−ξ||E0,
  for all *ϕ*, *ξ* ∈ *E*_0_;
(iv) if {*ϕ*_*n*_} is a sequence in *E*_0_ such that *ϕ*_*n*_ → *ϕ* as *n* → +*∞* and *α*(*ϕ*_*n*_(*c*), *ϕ*_*n*+1_(*c*)) ≥ *η*(*ϕ*_*n*_(*c*), *ϕ*_*n*+1_(*c*)) for all *n* ∈ *ℕ* ∪ 0, then *α*(*ϕ*_*n*_(*c*), *ϕ*(*c*)) ≥ *η*(*ϕ*_*n*_(*c*), *ϕ*(*c*)) for all *n* ∈ *ℕ* ∪ 0;
(v) there exists *ϕ*_0_ ∈ *ℛ*_*c*_ such that *α*(*ϕ*_0_(*c*), *Tϕ*_0_) ≥ *η*(*ϕ*_0_(*c*), *Tϕ*_0_).

Then, *T* has a *PPF* dependent fixed point ϕ* ∈ *ℛ*_*c*_.

**Proof**

Let *ϕ*_0_ ∈ *ℛ*_*c*_ such that *α*(*ϕ*_0_(*c*), *Tϕ*_0_) ≥ *η*(*ϕ*_0_(*c*), *Tϕ*_0_). Since *Tϕ*_0_ ∈ *E*, there exists *x*_1_ ∈ *E* such that *Tϕ*_0_ = *x*_1_. Choose *ϕ*_1_ ∈ *ℛ*_*c*_ such that

(20) $$\begin{array}{c} x_{1}=\phi_{1}\left(c\right). \end{array}$$

By continuing this process, by induction, we can build a sequence {*ϕ*_*n*_} in *ℛ*_*c*_⊆*E* such that,

(21) $$\begin{array}{c} T\phi_{n-1}=\phi_{n}\left(c\right),\;\forall n\in \mathbb{N}. \end{array}$$

Hence, from Lemma 12, we have

(22) α(ϕm(c),ϕn(c))≥η(ϕm(c),ϕn(c)),∀m,n∈ℕ  with  m<n.

Since *ℛ*_*c*_ is algebraically closed with respect to difference, it follows that

(23) $$\begin{array}{c} {||\phi_{n-1}-\phi_{n}||}_{E_{0}}={||\phi_{n-1}(c)-\phi_{n}(c)||}_{E},\;\forall n\in \mathbb{N}. \end{array}$$

Then, by (iii), we get

(24) ||ϕn−ϕn+1||E0=||ϕn(c)−ϕn+1(c)||E=||Tϕn−1−Tϕn||E≤β(||ϕn−1−ϕn||E0)||ϕn−1−ϕn||E0,

and so

(25) ||ϕn−ϕn+1||E0≤β(||ϕn−1−ϕn||E0)||ϕn−1−ϕn||E0<||ϕn−1−ϕn||E0,

for all *n* ∈ *ℕ*. This implies that the sequence {||*ϕ*_*n*_−*ϕ*_*n*+1_||_*E*_0__} is decreasing in ℝ_+_. Then, there exists *r* ≥ 0 such that lim⁡_*n*→+*∞*_||*ϕ*_*n*_−*ϕ*_*n*+1_||_*E*_0__ = *r*. Assume *r* > 0. Now, by taking limit as *n* → +*∞* in (24), we get

(26) $$\begin{array}{c} r\leq \underset{n\to +\infty }{\lim}\beta \left({||\phi_{n-1}-\phi_{n}||}_{E_{0}}\right)r, \end{array}$$

which implies 1 ≤ lim⁡_*n*→+*∞*_ *β*(||*ϕ*_*n*−1_−*ϕ*_*n*_||_*E*_0__). That is,

(27) $$\begin{array}{c} \underset{n\to +\infty }{\lim}\beta \left({||\phi_{n-1}-\phi_{n}||}_{E_{0}}\right)=1, \end{array}$$

and since *β* ∈ *ℱ*, lim⁡_*n*→+*∞*_||*ϕ*_*n*−1_−*ϕ*_*n*_||_*E*_0__ = 0 which is a contradiction. Hence, *r* = 0. That is,

(28) $$\begin{array}{c} \underset{n\to +\infty }{\lim}{||\phi_{n-1}-\phi_{n}||}_{E_{0}}=0. \end{array}$$

Now, we prove that the sequence {*ϕ*_*n*_} is Cauchy in *ℛ*_*c*_. Assume the contrary; then there exist *ɛ* > 0 and two sequences {*m*_*k*_} and {*n*_*k*_} with *k* ≤ *m*_*k*_ < *n*_*k*_ such that

(29) $$\begin{array}{c} {||\phi_{m_{k}}-\phi_{n_{k}}||}_{E_{0}}\geq ɛ,\;\;{||\phi_{m_{k}}-\phi_{n_{k}-1}||}_{E_{0}}<ɛ. \end{array}$$

From

(30) ɛ≤||ϕmk−ϕnk||E0≤||ϕmk−ϕnk−1||E0+||ϕnk−1−ϕnk||E0<ɛ+||ϕnk−1−ϕnk||E0,

letting *k* → +*∞*, we get

(31) $$\begin{array}{c} \underset{n\to +\infty }{\lim}{||\phi_{m_{k}}-\phi_{n_{k}}||}_{E_{0}}=ɛ. \end{array}$$

By triangle inequality, we have

(32) ||ϕmk−ϕnk||E0≤||ϕmk−ϕmk+1||E0+||ϕmk+1−ϕnk+1||E0+||ϕnk−ϕnk+1||E0.

On the other hand, by (iii) and (21), we have

(33) $$\begin{array}{c} {||\phi_{m_{k}+1}-\phi_{n_{k}+1}||}_{E_{0}}\leq \beta \left({||\phi_{m_{k}}-\phi_{n_{k}}||}_{E_{0}}\right){||\phi_{m_{k}}-\phi_{n_{k}}||}_{E_{0}}. \end{array}$$

Therefore, we get

(34) ||ϕmk−ϕnk||E0≤||ϕmk−ϕmk+1||E0+β(||ϕmk−ϕnk||E0)||ϕmk−ϕnk||E0+||ϕnk−ϕnk+1||E0,

which implies

(35) (1−β(||ϕmk−ϕnk||E0))||ϕmk−ϕnk||E0  ≤||ϕmk−ϕmk+1||E0+||ϕnk−ϕnk+1||E0.

Taking limit as *k* → +*∞* in the above inequality and applying (28) and (31), we get

(36) $$\begin{array}{c} \underset{k\to +\infty }{\lim}\left(1-\beta \left({||\phi_{m_{k}}-\phi_{n_{k}}||}_{E_{0}}\right)\right)=0, \end{array}$$

which implies lim⁡_*k*→+*∞*_ *β*(||*ϕ*_*m*_*k*__−*ϕ*_*n*_*k*__||_*E*_0__) = 1 and since *β* ∈ *ℱ*, we deduce

(37) $$\begin{array}{c} \underset{k\to +\infty }{\lim}{||\phi_{m_{k}}-\phi_{n_{k}}||}_{E_{0}}=0, \end{array}$$

which is a contradiction. Consequently

(38) $$\begin{array}{c} \underset{m,n\to +\infty }{\lim}{||\phi_{n}-\phi_{m}||}_{E_{0}}=0, \end{array}$$

and hence {*ϕ*_*n*_} is a Cauchy sequence in *ℛ*_*c*_⊆*E*_0_. By the completeness of *E*_0_ we get that {*ϕ*_*n*_} converges to a point ϕ* ∈ *E*_0_; that is, *ϕ*_*n*_ → ϕ* as *n* → +*∞*. Since *ℛ*_*c*_ is topologically closed, we deduce ϕ* ∈ *ℛ*_*c*_. From (iv) we have *α*(*ϕ*_*n*_(*c*), ϕ*(*c*)) ≥ *η*(*ϕ*_*n*_(*c*), ϕ*(*c*)) for all *n* ∈ *ℕ* ∪ 0. Then, from (iii) we get

(39) ||Tϕ∗−ϕ∗(c)||E≤||Tϕ∗−Tϕn||E+||Tϕn−ϕ∗(c)||E=||Tϕ∗−Tϕn||E+||ϕn+1(c)−ϕ∗(c)||E≤β(||ϕ∗−ϕn||E0)||ϕ∗−ϕn||E0+||ϕn+1(c)−ϕ∗(c)||E,

for all *n* ∈ *ℕ*. Taking limit as *n* → +*∞* in the above inequality, we get

(40) $$\begin{array}{c} {||T\phi^{*}-\phi^{*}(c)||}_{E}=0; \end{array}$$

that is,

(41) $$\begin{array}{c} T\phi^{*}=\phi^{*}\left(c\right), \end{array}$$

which implies that ϕ* is a PPF dependent fixed point of *T* in *ℛ*_*c*_.

If in Theorem 13 we take *η*(*ϕ*(*c*), *ξ*(*c*)) = 1 for all *ϕ*, *ξ* ∈ *E*_0_, then we deduce the following corollary.

**Corollary 14**

Let *T* : *E*_0_ → *E* and *α* : *E* × *E* → [0, +*∞*) be two mappings satisfying the following assertions:

(i) there exists *c* ∈ *I* such that *ℛ*_*c*_ is topologically closed and algebraically closed with respect to difference;
(ii) *T* is a triangular *α*_*c*_-admissible mapping;
(iii) there exists *β* ∈ *ℱ* such that
  (42) α(ϕ(c),ξ(c))≥1  ⟹||Tϕ−Tξ||E≤β(||ϕ−ξ||E0)||ϕ−ξ||E0,
  for all *ϕ*, *ξ* ∈ *E*_0_;
(iv) if {*ϕ*_*n*_} is a sequence in *E*_0_ such that *ϕ*_*n*_ → *ϕ* as *n* → +*∞* and *α*(*ϕ*_*n*_(*c*), *ϕ*_*n*+1_(*c*)) ≥ 1 for all *n* ∈ *ℕ* ∪ 0, then *α*(*ϕ*_*n*_(*c*), *ϕ*(*c*)) ≥ 1 for all *n* ∈ *ℕ* ∪ 0;
(v) there exists *ϕ*_0_ ∈ *ℛ*_*c*_ such that *α*(*ϕ*_0_(*c*), *Tϕ*_0_) ≥ 1.

Then, *T* has a *PPF* dependent fixed point ϕ* ∈ *ℛ*_*c*_.

If in Theorem 13 we take *α*(*ϕ*(*c*), *ξ*(*c*)) = 1 for all *ϕ*, *ξ* ∈ *E*_0_, then we deduce the following corollary.

**Corollary 15**

Let *T* : *E*_0_ → *E* and *η* : *E* × *E* → [0, +*∞*) be two mappings satisfying the following assertions:

(i) there exists *c* ∈ *I* such that *ℛ*_*c*_ is topologically closed and algebraically closed with respect to difference;
(ii) *T* is a triangular *η*_*c*_-subadmissible mapping;
(iii) there exists *β* ∈ *ℱ* such that
  (43) η(ϕ(c),ξ(c))≤1  ⟹||Tϕ−Tξ||E≤β(||ϕ−ξ||E0)||ϕ−ξ||E0,
  for all *ϕ*, *ξ* ∈ *E*_0_;
(iv) if {*ϕ*_*n*_} is a sequence in *E*_0_ such that *ϕ*_*n*_ → *ϕ* as *n* → +*∞* and *η*(*ϕ*_*n*_(*c*), *ϕ*_*n*+1_(*c*)) ≤ 1 for all *n* ∈ *ℕ* ∪ 0, then *η*(*ϕ*_*n*_(*c*), *ϕ*) ≤ 1 for all *n* ∈ *ℕ* ∪ 0;
(v) there exists *ϕ*_0_ ∈ *ℛ*_*c*_ such that *η*(*ϕ*_0_(*c*), *Tϕ*_0_) ≤ 1.

Then, *T* has a *PPF* dependent fixed point ϕ* ∈ *ℛ*_*c*_.

**Definition 16**

Let *c* ∈ *I* and *S* : *E*_0_ → *E*_0_, *T* : *E*_0_ → *E*, *α*, *η* : *E* × *E* → [0, +*∞*). We say that (*S*, *T*) is a triangular *α*_*c*_-admissible pair with respect to *η*_*c*_ if, for *ϕ*, *ξ*, *π* ∈ *E*_0_,

(ST1)α((Sϕ)(c),(Sξ)(c))≥η((Sϕ)(c),(Sξ)(c))  ⟹α(Tϕ,Tξ)≥η(Tϕ,Tξ),

(44) α((Sϕ)(c),(Sξ)(c))≥η((Sϕ)(c),(Sξ)(c)),α((Sξ)(c),(Sπ)(c))≥η((Sϕ)(c),(Sξ)(c))  ⟹α((Sϕ)(c),(Sπ)(c))≥η((Sϕ)(c),(Sπ)(c)).

Note that if we take *η*(*ϕ*(*c*), *ξ*(*c*)) = 1, then, we say that (*S*, *T*) is a triangular *α*_*c*_-admissible pair. Also, if we take *α*(*ϕ*(*c*), *ξ*(*c*)) = 1, then we say that (*S*, *T*) is a triangular *η*_*c*_-subadmissible pair.

The following theorem gives a result of existence of PPF dependent coincidence points.

**Theorem 17**

Let *S* : *E*_0_ → *E*_0_, *T* : *E*_0_ → *E*, and *α*, *η* : *E* × *E* → [0, +*∞*) be four mappings satisfying the following assertions:

(i) there exists *c* ∈ *I* such that *S*(*ℛ*_*c*_) ⊂ *ℛ*_*c*_ is algebraically closed with respect to difference;
(ii) (*S*, *T*) is a triangular *α*_*c*_-admissible pair with respect to *η*_*c*_;
(iii) there exists *β* ∈ *ℱ* such that
  (45) α((Sϕ)(c),(Sξ)(c))≥η((Sϕ)(c),(Sξ)(c))  ⟹||Tϕ−Tξ||E≤β(||Sϕ−Sξ||E0)||Sϕ−Sξ||E0,
  for all *ϕ*, *ξ* ∈ *ℛ*_*c*_;
(iv) if {*Sϕ*_*n*_} is a sequence in *ℛ*_*c*_ such that *Sϕ*_*n*_ → *Sϕ* as *n* → +*∞* and *α*((*Sϕ*_*n*_)(*c*), (*Sϕ*_*n*+1_)(*c*)) ≥ *η*((*Sϕ*_*n*_)(*c*), (*Sϕ*_*n*+1_)(*c*)) for all *n* ∈ *ℕ* ∪ 0, then *α*((*Sϕ*_*n*_)(*c*), (*Sϕ*)(*c*)) ≥ *η*((*Sϕ*_*n*_)(*c*), (*Sϕ*)(*c*)) for all *n* ∈ *ℕ* ∪ 0;
(v) there exists *ϕ*_0_ ∈ *ℛ*_*c*_ such that *α*((*Sϕ*_0_)(*c*), *Tϕ*_0_) ≥ *η*((*Sϕ*_0_)(*c*), *Tϕ*_0_);
(vi) *S*(*ℛ*_*c*_) is complete in *ℛ*_*c*_;
(vii) *T*(*ℛ*_*c*_)⊂{(*Sϕ*)(*c*) : *ϕ* ∈ *ℛ*_*c*_}.

Then, there exists ϕ* ∈ *ℛ*_*c*_ such that Sϕ* ∈ *ℛ*_*c*_ is a *PPF* dependent fixed point of *T* and hence ϕ* is a *PPF* dependent coincidence point of *S* and *T*.

**Proof**

Let *ϕ*_0_ ∈ *ℛ*_*c*_ such that *α*((*Sϕ*_0_)(*c*), *Tϕ*_0_) ≥ *η*((*Sϕ*_0_)(*c*), *Tϕ*_0_). By condition (vii), there exists *ϕ*_1_ ∈ *ℛ*_*c*_ such that

(46) $$\begin{array}{c} T\phi_{0}=\left(S\phi_{1}\right)\left(c\right). \end{array}$$

By continuing this process, by induction, we can build a sequence {*ϕ*_*n*_} in *ℛ*_*c*_ such that

(47) $$\begin{array}{c} T\phi_{n-1}=\left(S\phi_{n}\right)\left(c\right),\;\forall n\in \mathbb{N}. \end{array}$$

Hence, from Lemma 12, we have

(48) α((Sϕm)(c),(Sϕn)(c))≥η((Sϕm)(c),(Sϕn)(c)),∀m,n∈ℕ  with  m<n.

Since *S*(*ℛ*_*c*_) is algebraically closed with respect to difference, it follows that

(49) $$\begin{array}{c} {||S\phi_{n-1}-S\phi_{n}||}_{E_{0}}={||\left(S\phi_{n-1})(c)-(S\phi_{n})(c\right)||}_{E},\;\forall n\in \mathbb{N}. \end{array}$$

Then, by (iii), we get

(50) ||Sϕn−Sϕn+1||E0=||(Sϕn)(c)−(Sϕn+1)(c)||E=||Tϕn−1−Tϕn||E≤β(||Sϕn−1−Sϕn||E0)||Sϕn−1−Sϕn||E0,

and so

(51) ||Sϕn−Sϕn+1||E0≤β(||Sϕn−1−Sϕn||E0)||Sϕn−1−Sϕn||E0<||Sϕn−1−Sϕn||E0,

for all *n* ∈ *ℕ*. This implies that the sequence {||*Sϕ*_*n*_−*Sϕ*_*n*+1_||_*E*_0__} is decreasing in ℝ_+_. Then, there exists *r* ≥ 0 such that lim⁡_*n*→+*∞*_||*Sϕ*_*n*_−*Sϕ*_*n*+1_||_*E*_0__ = *r*. Assume *r* > 0. Now by taking limit as *n* → +*∞* in (50) we get

(52) $$\begin{array}{c} r\leq \underset{n\to +\infty }{\lim}\beta \left({||S\phi_{n-1}-S\phi_{n}||}_{E_{0}}\right)r, \end{array}$$

which implies 1 ≤ lim⁡_*n*→+*∞*_ *β*(||*Sϕ*_*n*−1_−*Sϕ*_*n*_||_*E*_0__). That is,

(53) $$\begin{array}{c} \underset{n\to +\infty }{\lim}\beta \left({||S\phi_{n-1}-S\phi_{n}||}_{E_{0}}\right)=1, \end{array}$$

and since *β* ∈ *ℱ*, lim⁡_*n*→+*∞*_||*Sϕ*_*n*−1_−*Sϕ*_*n*_||_*E*_0__ = 0 which is a contradiction. Hence, *r* = 0. That is,

(54) $$\begin{array}{c} \underset{n\to +\infty }{\lim}{||S\phi_{n-1}-S\phi_{n}||}_{E_{0}}=0. \end{array}$$

Now, we prove that the sequence {*Sϕ*_*n*_} is Cauchy in *S*(*ℛ*_*c*_). Assume the contrary; then there exist *ɛ* > 0 and two sequences {*m*_*k*_} and {*n*_*k*_} with *k* ≤ *m*_*k*_ < *n*_*k*_ such that

(55) $$\begin{array}{c} {||S\phi_{m_{k}}-S\phi_{n_{k}}||}_{E_{0}}\geq ɛ,\;\;{||S\phi_{m_{k}}-S\phi_{n_{k}-1}||}_{E_{0}}<ɛ. \end{array}$$

From

(56) ɛ≤||Sϕmk−Sϕnk||E0≤||Sϕmk−Sϕnk−1||E0+||Sϕnk−1−Sϕnk||E0<ɛ+||Sϕnk−1−Sϕnk||E0,

letting *k* → +*∞*, we get

(57) $$\begin{array}{c} \underset{n\to +\infty }{\lim}{||S\phi_{m_{k}}-S\phi_{n_{k}}||}_{E_{0}}=ɛ. \end{array}$$

By triangle inequality, we have

(58) ||Sϕmk−Sϕnk||E0≤||Sϕmk−Sϕmk+1||E0+||Sϕmk+1−Sϕnk+1||E0+||Sϕnk−Sϕnk+1||E0.

On the other hand, by (iii) and (47), we have

(59) $$\begin{array}{c} {||S\phi_{m_{k}+1}-S\phi_{n_{k}+1}||}_{E_{0}}\leq \beta \left({||S\phi_{m_{k}}-S\phi_{n_{k}}||}_{E_{0}}\right){||S\phi_{m_{k}}-S\phi_{n_{k}}||}_{E_{0}}. \end{array}$$

Therefore, we get

(60) ||Sϕmk−Sϕnk||E0≤||Sϕmk−Sϕmk+1||E0+β(||Sϕmk−Sϕnk||E0)||Sϕmk−Sϕnk||E0+||Sϕnk−Sϕnk+1||E0,

which implies

(61) (1−β(||Sϕmk−Sϕnk||E0))||Sϕmk−Sϕnk||E0  ≤||Sϕmk−Sϕmk+1||E0+||Sϕnk−Sϕnk+1||E0.

Taking limit as *k* → +*∞* in the above inequality and applying (54) and (57), we get

(62) $$\begin{array}{c} \underset{k\to +\infty }{\lim}\left(1-\beta \left({||S\phi_{m_{k}}-S\phi_{n_{k}}||}_{E_{0}}\right)\right)=0, \end{array}$$

which implies lim⁡_*k*→+*∞*_ *β*(||*Sϕ*_*m*_*k*__−*Sϕ*_*n*_*k*__||_*E*_0__) = 1 and since *β* ∈ *ℱ*, we deduce

(63) $$\begin{array}{c} \underset{k\to +\infty }{\lim}{||S\phi_{m_{k}}-S\phi_{n_{k}}||}_{E_{0}}=0, \end{array}$$

which is a contradiction. Consequently

(64) $$\begin{array}{c} \underset{m,n\to +\infty }{\lim}{||S\phi_{n}-S\phi_{m}||}_{E_{0}}=0, \end{array}$$

and hence {*Sϕ*_*n*_} is a Cauchy sequence in *S*(*ℛ*_*c*_) ⊂ *ℛ*_*c*_. By the completeness of *S*(*ℛ*_*c*_), there exists ϕ* ∈ *ℛ*_*c*_ such that *Sϕ*_*n*_ → Sϕ* as *n* → +*∞*. From (iv), we have *α*((*Sϕ*_*n*_)(*c*), (Sϕ*)(*c*)) ≥ *η*((*Sϕ*_*n*_)(*c*), (Sϕ*)(*c*)) for all *n* ∈ *ℕ* ∪ 0. Then from (iii) we get

(65) ||Tϕ∗−(Sϕ∗)(c)||E  ≤||Tϕ∗−Tϕn||E+||Tϕn−(Sϕ∗)(c)||E  =||Tϕ∗−Tϕn||E+||(Sϕn+1)(c)−(Sϕ∗)(c)||E  ≤β(||Sϕ∗−Sϕn||E0)||Sϕ∗−Sϕn||E0   +||(Sϕn+1)(c)−(Sϕ∗)(c)||E,

for all *n* ∈ *ℕ*. Taking limit as *n* → +*∞* in the above inequality, we get

(66) $$\begin{array}{c} {||T\phi^{*}-(S\phi^{*})(c)||}_{E}=0. \end{array}$$

That is,

(67) $$\begin{array}{c} T\phi^{*}=\left(S\phi^{*}\right)\left(c\right), \end{array}$$

which implies that Sϕ* is a PPF dependent fixed point of *T* in *S*(*ℛ*_*c*_) and hence ϕ* is a PPF dependent coincidence point of *S* and *T*.

If in Theorem 17 we take *η*(*ϕ*(*c*), *ξ*(*c*)) = 1 for all *ϕ*, *ξ* ∈ *E*_0_, then we deduce the following corollary.

**Corollary 18**

Let *S* : *E*_0_ → *E*_0_, *T* : *E*_0_ → *E*, and *α* : *E* × *E* → [0, +*∞*) be three mappings satisfying the following assertions:

(i) there exists *c* ∈ *I* such that *S*(*ℛ*_*c*_) ⊂ *ℛ*_*c*_ is algebraically closed with respect to difference;
(ii) (*S*, *T*) is a triangular *α*_*c*_-admissible pair;
(iii) there exists *β* ∈ *ℱ* such that
  (68) α((Sϕ)(c),(Sξ)(c))≥1  ⟹||Tϕ−Tξ||E≤β(||Sϕ−Sξ||E0)||Sϕ−Sξ||E0,
  for all *ϕ*, *ξ* ∈ *ℛ*_*c*_;
(iv) if {*Sϕ*_*n*_} is a sequence in *ℛ*_*c*_ such that *Sϕ*_*n*_ → *Sϕ* as *n* → +*∞* and *α*((*Sϕ*_*n*_)(*c*), (*Sϕ*_*n*+1_)(*c*)) ≥ 1 for all *n* ∈ *ℕ* ∪ 0, then *α*((*Sϕ*_*n*_)(*c*), (*Sϕ*)(*c*)) ≥ 1 for all *n* ∈ *ℕ* ∪ 0;
(v) there exists *ϕ*_0_ ∈ *ℛ*_*c*_ such that *α*((*Sϕ*_0_)(*c*), *Tϕ*_0_) ≥ 1;
(vi) *S*(*ℛ*_*c*_) is complete in *ℛ*_*c*_;
(vii) *T*(*ℛ*_*c*_)⊂{(*Sϕ*)(*c*) : *ϕ* ∈ *ℛ*_*c*_}.

Then, *S* and *T* have a *PPF* dependent coincidence point ϕ* ∈ *ℛ*_*c*_.

If in Theorem 13 we take *α*(*ϕ*(*c*), *ξ*(*c*)) = 1 for all *ϕ*, *ξ* ∈ *E*_0_, then we deduce the following corollary.

**Corollary 19**

Let *S* : *E*_0_ → *E*_0_, *T* : *E*_0_ → *E*, and *η* : *E* × *E* → [0, +*∞*) be three mappings satisfying the following assertions:

(i) there exists *c* ∈ *I* such that *S*(*ℛ*_*c*_) ⊂ *ℛ*_*c*_ is algebraically closed with respect to difference;
(ii) (*S*, *T*) is a triangular *α*_*c*_-subadmissible pair;
(iii) there exists *β* ∈ *ℱ* such that
  (69) η((Sϕ)(c),(Sξ)(c))≤1  ⟹||Tϕ−Tξ||E≤β(||Sϕ−Sξ||E0)||Sϕ−Sξ||E0,
  for all *ϕ*, *ξ* ∈ *ℛ*_*c*_;
(iv) if {*Sϕ*_*n*_} is a sequence in *E*_0_ such that *Sϕ*_*n*_ → *Sϕ* as *n* → +*∞* and *η*((*Sϕ*_*n*_)(*c*), (*Sϕ*_*n*+1_)(*c*)) ≤ 1 for all *n* ∈ *ℕ* ∪ 0, then *η*((*Sϕ*_*n*_)(*c*), (*Sϕ*)(*c*)) ≤ 1;
(v) there exists *ϕ*_0_ ∈ *ℛ*_*c*_ such that *η*((*Sϕ*_0_)(*c*), *Tϕ*_0_) ≤ 1;
(vi) *S*(*ℛ*_*c*_) is complete in *ℛ*_*c*_;
(vii) *T*(*ℛ*_*c*_)⊂{(*Sϕ*)(*c*) : *ϕ* ∈ *ℛ*_*c*_}.

Then, *S* and *T* have a *PPF* dependent coincidence point ϕ* ∈ *ℛ*_*c*_.

### 2.1. Consequences of Corollary 14

**Theorem 20**

Let *T* : *E*_0_ → *E* and *α* : *E* × *E* → [0, +*∞*) be two mappings satisfying the following assertions:

(i) there exists *c* ∈ *I* such that *ℛ*_*c*_ is topologically closed and algebraically closed with respect to difference;
(ii) *T* is a triangular *α*_*c*_-admissible mapping;
(iii) there exists *β* ∈ *ℱ* such that
  (70) $$\begin{array}{c} \alpha \left(\phi \left(c\right),\xi \left(c\right)\right){||T\phi -T\xi ||}_{E}\leq \beta \left({||\phi -\xi ||}_{E_{0}}\right){||\phi -\xi ||}_{E_{0}}, \end{array}$$
  for all *ϕ*, *ξ* ∈ *E*_0_;
(iv) if {*ϕ*_*n*_} is a sequence in *E*_0_ such that *ϕ*_*n*_ → *ϕ* as *n* → +*∞* and *α*(*ϕ*_*n*_(*c*), *ϕ*_*n*+1_(*c*)) ≥ 1 for all *n* ∈ *ℕ* ∪ 0, then *α*(*ϕ*_*n*_(*c*), *ϕ*(*c*)) ≥ 1 for all *n* ∈ *ℕ* ∪ 0;
(v) there exists *ϕ*_0_ ∈ *ℛ*_*c*_ such that *α*(*ϕ*_0_(*c*), *Tϕ*_0_) ≥ 1.

Then, *T* has a *PPF* dependent fixed point ϕ* ∈ *ℛ*_*c*_.

**Proof**

Let *α*(*ϕ*(*c*), *ξ*(*c*)) ≥ 1; then by (iii) we have

(71) ||Tϕ−Tξ||E≤α(ϕ(c),ξ(c))||Tϕ−Tξ||E≤β(||ϕ−ξ||E0)||ϕ−ξ||E0.

That is, all conditions of Corollary 14 hold and *T* has a PPF dependent fixed point ϕ* ∈ *ℛ*_*c*_.

**Theorem 21**

Let *T* : *E*_0_ → *E* and *α* : *E* × *E* → [0, +*∞*) be two mappings satisfying the following assertions:

(i) there exists *c* ∈ *I* such that *ℛ*_*c*_ is topologically closed and algebraically closed with respect to difference;
(ii) *T* is a triangular *α*_*c*_-admissible mapping;
(iii) there exists *β* ∈ *ℱ* such that
  (72) $$\begin{array}{c} {\left({||T\phi -T\xi ||}_{E}+\epsilon \right)}^{\alpha (\phi (c),\xi (c))}\leq \beta \left({||\phi -\xi ||}_{E_{0}}\right){||\phi -\xi ||}_{E_{0}}+\epsilon , \end{array}$$
  for all *ϕ*, *ξ* ∈ *E*_0_, where *ϵ* ≥ 1;
(iv) if {*ϕ*_*n*_} is a sequence in *E*_0_ such that *ϕ*_*n*_ → *ϕ* as *n* → +*∞* and *α*(*ϕ*_*n*_(*c*), *ϕ*_*n*+1_(*c*)) ≥ 1 for all *n* ∈ *ℕ* ∪ 0, then *α*(*ϕ*_*n*_(*c*), *ϕ*(*c*)) ≥ 1 for all *n* ∈ *ℕ* ∪ 0;
(v) there exists *ϕ*_0_ ∈ *ℛ*_*c*_ such that *α*(*ϕ*_0_(*c*), *Tϕ*_0_) ≥ 1.

Then, *T* has a *PPF* dependent fixed point ϕ* ∈ *ℛ*_*c*_.

**Proof**

Let *α*(*ϕ*(*c*), *ξ*(*c*)) ≥ 1; then by (iii) we have

(73) ||Tϕ−Tξ||E+ϵ≤(||Tϕ−Tξ||E+ϵ)α(ϕ(c),ξ(c))≤β(||ϕ−ξ||E0)||ϕ−ξ||E0+ϵ,

which implies ||*Tϕ*−*Tξ*||_*E*_ ≤ *β*(||*ϕ*−*ξ*||_*E*_0__)||*ϕ*−*ξ*||_*E*_0__. That is, all conditions of Corollary 14 hold and *T* has a PPF dependent fixed point ϕ* ∈ *ℛ*_*c*_.

**Theorem 22**

Let *T* : *E*_0_ → *E* and *α* : *E* × *E* → [0, +*∞*) be two mappings satisfying the following assertions:

(i) there exists *c* ∈ *I* such that *ℛ*_*c*_ is topologically closed and algebraically closed with respect to difference;
(ii) *T* is a triangular *α*_*c*_-admissible mapping;
(iii) there exists *β* ∈ *ℱ* such that
  (74) $$\begin{array}{c} {\left(\alpha \left(\phi \left(c\right),\xi \left(c\right)\right)-1+\sigma \right)}^{{||T\phi -T\xi ||}_{E}}\leq \epsilon^{\beta ({||\phi -\xi ||}_{E_{0}}){||\phi -\xi ||}_{E_{0}}}, \end{array}$$
  for all *ϕ*, *ξ* ∈ *E*_0_, where 1 < *ϵ* ≤ *σ*;
(iv) if {*ϕ*_*n*_} is a sequence in *E*_0_ such that *ϕ*_*n*_ → *ϕ* as *n* → +*∞* and *α*(*ϕ*_*n*_(*c*), *ϕ*_*n*+1_(*c*)) ≥ 1 for all *n* ∈ *ℕ* ∪ 0, then *α*(*ϕ*_*n*_(*c*), *ϕ*(*c*)) ≥ 1 for all *n* ∈ *ℕ* ∪ 0;
(v) there exists *ϕ*_0_ ∈ *ℛ*_*c*_ such that *α*(*ϕ*_0_(*c*), *Tϕ*_0_) ≥ 1.

Then, *T* has a *PPF* dependent fixed point ϕ* ∈ *ℛ*_*c*_.

**Proof**

Let *α*(*ϕ*(*c*), *ξ*(*c*)) ≥ 1; then by (iii) we have

(75) σ||Tϕ−Tξ||E≤(α(ϕ(c),ξ(c))−1+σ)||Tϕ−Tξ||E≤ϵβ(||ϕ−ξ||E0)||ϕ−ξ||E0≤σβ(||ϕ−ξ||E0)||ϕ−ξ||E0,

which implies ||*Tϕ*−*Tξ*||_*E*_ ≤ *β*(||*ϕ*−*ξ*||_*E*_0__)||*ϕ*−*ξ*||_*E*_0__. That is, all conditions of Corollary 14 hold and *T* has a PPF dependent fixed point ϕ* ∈ *ℛ*_*c*_.

### 2.2. Consequences of Corollary 15

**Theorem 23**

Let *T* : *E*_0_ → *E* and *η* : *E* × *E* → [0, +*∞*) be two mappings satisfying the following assertions:

(i) there exists *c* ∈ *I* such that *ℛ*_*c*_ is topologically closed and algebraically closed with respect to difference;
(ii) *T* is a triangular *η*_*c*_-subadmissible mapping;
(iii) there exists *β* ∈ *ℱ* such that
  (76) $$\begin{array}{c} {||T\phi -T\xi ||}_{E}\leq \eta \left(\phi \left(c\right),\xi \left(c\right)\right)\beta \left({||\phi -\xi ||}_{E_{0}}\right){||\phi -\xi ||}_{E_{0}}, \end{array}$$
  for all *ϕ*, *ξ* ∈ *E*_0_;
(iv) if {*ϕ*_*n*_} is a sequence in *E*_0_ such that *ϕ*_*n*_ → *ϕ* as *n* → *∞* and *η*(*ϕ*_*n*_(*c*), *ϕ*_*n*+1_(*c*)) ≤ 1 for all *n* ∈ *ℕ* ∪ 0, then *η*(*ϕ*_*n*_(*c*), *ϕ*(*c*)) ≤ 1 for all *n* ∈ *ℕ* ∪ 0;
(v) there exists *ϕ*_0_ ∈ *ℛ*_*c*_ such that *η*(*ϕ*_0_(*c*), *Tϕ*_0_) ≤ 1.

Then, *T* has a *PPF* dependent fixed point ϕ* ∈ *ℛ*_*c*_.

**Theorem 24**

Let *T* : *E*_0_ → *E* and *η* : *E* × *E* → [0, *∞*) be two mappings satisfying the following assertions:

(i) there exists *c* ∈ *I* such that *ℛ*_*c*_ is topologically closed and algebraically closed with respect to difference;
(ii) *T* is a triangular *η*_*c*_-subadmissible mapping;
(iii) there exists *β* ∈ *ℱ* such that
  (77) $$\begin{array}{c} {||T\phi -T\xi ||}_{E}+\epsilon \leq {\left(\beta \left({||\phi -\xi ||}_{E_{0}}\right){||\phi -\xi ||}_{E_{0}}+\epsilon \right)}^{\eta (\phi (c),\xi (c))}, \end{array}$$
  for all *ϕ*, *ξ* ∈ *E*_0_, where *ϵ* ≥ 1 and *ψ* ∈ Ψ;
(iv) if {*ϕ*_*n*_} is a sequence in *E*_0_ such that *ϕ*_*n*_ → *ϕ* as *n* → +*∞* and *η*(*ϕ*_*n*_(*c*), *ϕ*_*n*+1_(*c*)) ≤ 1 for all *n* ∈ *ℕ* ∪ 0, then *η*(*ϕ*_*n*_(*c*), *ϕ*(*c*)) ≤ 1 for all *n* ∈ *ℕ* ∪ 0;
(v) there exists *ϕ*_0_ ∈ *ℛ*_*c*_ such that *η*(*ϕ*_0_(*c*), *Tϕ*_0_) ≤ 1.

Then, *T* has a *PPF* dependent fixed point ϕ* ∈ *ℛ*_*c*_.

**Theorem 25**

Let *T* : *E*_0_ → *E* and *η* : *E* × *E* → [0, +*∞*) be two mappings satisfying the following assertions:

(i) there exists *c* ∈ *I* such that *ℛ*_*c*_ is topologically closed and algebraically closed with respect to difference;
(ii) *T* is a triangular *η*_*c*_-subadmissible mapping;
(iii) there exists *β* ∈ *ℱ* such that
  (78) $$\begin{array}{c} \sigma^{{||T\phi -T\xi ||}_{E}}\leq {\left(\eta \left(\phi \left(c\right),\xi \left(c\right)\right)+\epsilon -1\right)}^{\beta ({||\phi -\xi ||}_{E_{0}}){||\phi -\xi ||}_{E_{0}}}, \end{array}$$
  for all *ϕ*, *ξ* ∈ *E*_0_, where 1 < *ϵ* ≤ *σ* and *ψ* ∈ Ψ;
(iv) if {*ϕ*_*n*_} is a sequence in *E*_0_ such that *ϕ*_*n*_ → *ϕ* as *n* → +*∞* and *η*(*ϕ*_*n*_(*c*), *ϕ*_*n*+1_(*c*)) ≤ 1 for all *n* ∈ *ℕ* ∪ 0, then *η*(*ϕ*_*n*_(*c*), *ϕ*(*c*)) ≤ 1 for all *n* ∈ *ℕ* ∪ 0;
(v) there exists *ϕ*_0_ ∈ *ℛ*_*c*_ such that *η*(*ϕ*_0_(*c*), *Tϕ*_0_) ≤ 1.

Then, *T* has a *PPF* dependent fixed point ϕ* ∈ *ℛ*_*c*_.

### 2.3. Consequences of Corollary 18

**Theorem 26**

Let *S* : *E*_0_ → *E*_0_, *T* : *E*_0_ → *E*, and *α* : *E* × *E* → [0, +*∞*) be three mappings satisfying the following assertions:

(i) there exists *c* ∈ *I* such that *S*(*ℛ*_*c*_) ⊂ *ℛ*_*c*_ is algebraically closed with respect to difference;
(ii) (*S*, *T*) is a triangular *α*_*c*_-admissible pair;
(iii) there exists *β* ∈ *ℱ* such that
  (79) α((Sϕ)(c),(Sξ)(c))||Tϕ−Tξ||E  ≤β(||Sϕ−Sξ||E0)||Sϕ−Sξ||E0,
  for all *ϕ*, *ξ* ∈ *ℛ*_*c*_;
(iv) if {*Sϕ*_*n*_} is a sequence in *ℛ*_*c*_ such that *Sϕ*_*n*_ → *Sϕ* as *n* → +*∞* and *α*((*Sϕ*_*n*_)(*c*), (*Sϕ*_*n*+1_)(*c*)) ≥ 1 for all *n* ∈ *ℕ* ∪ 0, then *α*((*Sϕ*_*n*_)(*c*), (*Sϕ*)(*c*)) ≥ 1 for all *n* ∈ *ℕ* ∪ 0;
(v) there exists *ϕ*_0_ ∈ *ℛ*_*c*_ such that *α*((*Sϕ*_0_)(*c*), *Tϕ*_0_) ≥ 1;
(vi) *S*(*ℛ*_*c*_) is complete in *ℛ*_*c*_;
(vii) *T*(*ℛ*_*c*_)⊂{(*Sϕ*)(*c*) : *ϕ* ∈ *ℛ*_*c*_}.

Then, *S* and *T* have a *PPF* dependent coincidence point ϕ* ∈ *ℛ*_*c*_.

**Theorem 27**

Let *S* : *E*_0_ → *E*_0_, *T* : *E*_0_ → *E*, and *α* : *E* × *E* → [0, +*∞*) be three mappings satisfying the following assertions:

(i) there exists *c* ∈ *I* such that *S*(*ℛ*_*c*_) ⊂ *ℛ*_*c*_ is algebraically closed with respect to difference;
(ii) (*S*, *T*) is a triangular *α*_*c*_-admissible pair;
(iii) there exists *β* ∈ *ℱ* such that
  (80) (||Tϕ−Tξ||E+ϵ)α((Sϕ)(c),(Sξ)(c))  ≤β(||Sϕ−Sξ||E0)||Sϕ−Sξ||E0+ϵ,
  for all *ϕ*, *ξ* ∈ *ℛ*_*c*_ where *ϵ* ≥ 1;
(iv) if {*Sϕ*_*n*_} is a sequence in *E*_0_ such that *Sϕ*_*n*_ → *Sϕ* as *n* → +*∞* and *α*((*Sϕ*_*n*_)(*c*), (*Sϕ*_*n*+1_)(*c*)) ≥ 1 for all *n* ∈ *ℕ* ∪ 0, then *α*((*Sϕ*_*n*_)(*c*), (*Sϕ*)(*c*)) ≥ 1 for all *n* ∈ *ℕ* ∪ 0;
(v) there exists *ϕ*_0_ ∈ *ℛ*_*c*_ such that *α*((*Sϕ*_0_)(*c*), *Tϕ*_0_) ≥ 1;
(vi) *S*(*ℛ*_*c*_) is complete in *ℛ*_*c*_;
(vii) *T*(*ℛ*_*c*_)⊂{(*Sϕ*)(*c*) : *ϕ* ∈ *ℛ*_*c*_}.

Then, *S* and *T* have a *PPF* dependent coincidence point ϕ* ∈ *ℛ*_*c*_.

**Theorem 28**

Let *S* : *E*_0_ → *E*_0_, *T* : *E*_0_ → *E*, and *α* : *E* × *E* → [0, +*∞*) be three mappings satisfying the following assertions:

(i) there exists *c* ∈ *I* such that *S*(*ℛ*_*c*_) ⊂ *ℛ*_*c*_ is topologically closed and algebraically closed with respect to difference;
(ii) (*S*, *T*) is a triangular *α*_*c*_-admissible pair;
(iii) there exists *β* ∈ *ℱ* such that
  (81) (α((Sϕ)(c),(Sξ)(c))−1+σ)||Tϕ−Tξ||E  ≤ϵβ(||Sϕ−Sξ||E0)||Sϕ−Sξ||E0
  for all *ϕ*, *ξ* ∈ *ℛ*_*c*_, where 1 < *ϵ* ≤ *σ*;
(iv) if {*Sϕ*_*n*_} is a sequence in *E*_0_ such that *Sϕ*_*n*_ → *Sϕ* as *n* → +*∞* and *α*((*Sϕ*_*n*_)(*c*), (*Sϕ*_*n*+1_)(*c*)) ≥ 1 for all *n* ∈ *ℕ* ∪ 0, then *α*((*Sϕ*_*n*_)(*c*), (*Sϕ*)(*c*)) ≥ 1 for all *n* ∈ *ℕ* ∪ 0;
(v) there exists *ϕ*_0_ ∈ *ℛ*_*c*_ such that *α*((*Sϕ*_0_)(*c*), *Tϕ*_0_) ≥ 1;
(vi) *S*(*ℛ*_*c*_) is complete in *ℛ*_*c*_;
(vii) *T*(*ℛ*_*c*_)⊂{(*Sϕ*)(*c*) : *ϕ* ∈ *ℛ*_*c*_}.

Then, *S* and *T* have a *PPF* dependent coincidence point ϕ* ∈ *ℛ*_*c*_.

### 2.4. Consequences of Corollary 19

**Corollary 29**

Let *S* : *E*_0_ → *E*_0_, *T* : *E*_0_ → *E*, and *η* : *E* × *E* → [0, +*∞*) be three mappings satisfying the following assertions:

(i) there exists *c* ∈ *I* such that *S*(*ℛ*_*c*_) ⊂ *ℛ*_*c*_ is algebraically closed with respect to difference;
(ii) (*S*, *T*) is a triangular *α*_*c*_-subadmissible pair;
(iii) there exists *β* ∈ *ℱ* such that
  (82) ||Tϕ−Tξ||E  ≤η((Sϕ)(c),(Sξ)(c))β(||Sϕ−Sξ||E0)||Sϕ−Sξ||E0,
  for all *ϕ*, *ξ* ∈ *ℛ*_*c*_;
(iv) if {*Sϕ*_*n*_} is a sequence in *E*_0_ such that *Sϕ*_*n*_ → *Sϕ* as *n* → +*∞* and *η*((*Sϕ*_*n*_)(*c*), (*Sϕ*_*n*+1_)(*c*)) ≤ 1 for all *n* ∈ *ℕ* ∪ 0, then *η*((*Sϕ*_*n*_)(*c*), (*Sϕ*)(*c*)) ≤ 1 for all *n* ∈ *ℕ* ∪ 0;
(v) there exists *ϕ*_0_ ∈ *ℛ*_*c*_ such that *η*((*Sϕ*_0_)(*c*), *Tϕ*_0_) ≤ 1;
(vi) *S*(*ℛ*_*c*_) is complete in *ℛ*_*c*_;
(vii) *T*(*ℛ*_*c*_)⊂{(*Sϕ*)(*c*) : *ϕ* ∈ *ℛ*_*c*_}.

Then, *S* and *T* have a *PPF* dependent coincidence point ϕ* ∈ *ℛ*_*c*_.

**Corollary 30**

Let *S* : *E*_0_ → *E*_0_, *T* : *E*_0_ → *E*, and *η* : *E* × *E* → [0, +*∞*) be three mappings satisfying the following assertions:

(i) there exists *c* ∈ *I* such that *S*(*ℛ*_*c*_) ⊂ *ℛ*_*c*_ is algebraically closed with respect to difference;
(ii) (*S*, *T*) is a triangular *α*_*c*_-subadmissible pair;
(iii) there exists *β* ∈ *ℱ* such that
  (83) ||Tϕ−Tξ||E+ϵ  ≤(β(||Sϕ−Sξ||E0)||Sϕ−Sξ||E0+ϵ)η((Sϕ)(c),(Sξ)(c))
  for all *ϕ*, *ξ* ∈ *ℛ*_*c*_, where *ϵ* ≥ 1;
(iv) if {*Sϕ*_*n*_} is a sequence in *E*_0_ such that *Sϕ*_*n*_ → *Sϕ* as *n* → +*∞* and *η*((*Sϕ*_*n*_)(*c*), (*Sϕ*_*n*+1_)(*c*)) ≤ 1 for all *n* ∈ *ℕ* ∪ 0, then *η*((*Sϕ*_*n*_)(*c*), (*Sϕ*)(*c*)) ≤ 1 for all *n* ∈ *ℕ* ∪ 0;
(v) there exists *ϕ*_0_ ∈ *ℛ*_*c*_ such that *η*((*Sϕ*_0_)(*c*), *Tϕ*_0_) ≤ 1;
(vi) *S*(*ℛ*_*c*_) is complete in *ℛ*_*c*_;
(vii) *T*(*ℛ*_*c*_)⊂{(*Sϕ*)(*c*) : *ϕ* ∈ *ℛ*_*c*_}.

Then, *S* and *T* have a *PPF* dependent coincidence point ϕ* ∈ *ℛ*_*c*_.

**Corollary 31**

Let *S* : *E*_0_ → *E*_0_, *T* : *E*_0_ → *E*, and *η* : *E* × *E* → [0, +*∞*) be three mappings satisfying the following assertions:

(i) there exists *c* ∈ *I* such that *S*(*ℛ*_*c*_) ⊂ *ℛ*_*c*_ is topologically closed and algebraically closed with respect to difference;
(ii) (*S*, *T*) is a triangular *α*_*c*_-subadmissible pair;
(iii) there exists *β* ∈ *ℱ* such that
  (84) $$\begin{array}{c} \sigma^{{||T\phi -T\xi ||}_{E}}\leq {\left(\eta \left(\left(S\phi \right)\left(c\right),\left(S\xi \right)\left(c\right)\right)+\epsilon -1\right)}^{\beta ({||S\phi -S\xi ||}_{E_{0}}){||S\phi -S\xi ||}_{E_{0}}} \end{array}$$
  for all *ϕ*, *ξ* ∈ *ℛ*_*c*_, where 1 < *ϵ* ≤ *σ*;
(iv) if {*Sϕ*_*n*_} is a sequence in *E*_0_ such that *Sϕ*_*n*_ → *Sϕ* as *n* → +*∞* and *η*((*Sϕ*_*n*_)(*c*), (*Sϕ*_*n*+1_)(*c*)) ≤ 1 for all *n* ∈ *ℕ* ∪ 0, then *η*((*Sϕ*_*n*_)(*c*), (*Sϕ*)(*c*)) ≤ 1 for all *n* ∈ *ℕ* ∪ 0;
(v) there exists *ϕ*_0_ ∈ *ℛ*_*c*_ such that *η*((*Sϕ*_0_)(*c*), *Tϕ*_0_) ≤ 1;
(vi) *S*(*ℛ*_*c*_) is complete in *ℛ*_*c*_;
(vii) *T*(*ℛ*_*c*_)⊂{(*Sϕ*)(*c*) : *ϕ* ∈ *ℛ*_*c*_}.

Then, *S* and *T* have a *PPF* dependent coincidence point ϕ* ∈ *ℛ*_*c*_.
